# Photosynthetic acclimation responses of maize seedlings grown under artificial laboratory light gradients mimicking natural canopy conditions

**DOI:** 10.3389/fpls.2013.00334

**Published:** 2013-09-12

**Authors:** Matthias Hirth, Lars Dietzel, Sebastian Steiner, Robert Ludwig, Hannah Weidenbach, Jeannette Pfalz and, Thomas Pfannschmidt

**Affiliations:** Institut für Allgemeine Botanik und Pflanzenphysiologie, Lehrstuhl für Pflanzenphysiologie, Friedrich-Schiller-Universität Jena Jena, Germany

**Keywords:** photosynthesis, redox regulation, light quality, light acclimation, maize fields

## Abstract

In this study we assessed the ability of the C4 plant maize to perform long-term photosynthetic acclimation in an artificial light quality system previously used for analyzing short-term and long-term acclimation responses (LTR) in C3 plants. We aimed to test if this light system could be used as a tool for analyzing redox-regulated acclimation processes in maize seedlings. Photosynthetic parameters obtained from maize samples harvested in the field were used as control. The results indicated that field grown maize performed a pronounced LTR with significant differences between the top and the bottom levels of the plant stand corresponding to the strong light gradients occurring in it. We compared these data to results obtained from maize seedlings grown under artificial light sources preferentially exciting either photosystem II or photosystem I. In C3 plants, this light system induces redox signals within the photosynthetic electron transport chain which trigger state transitions and differential phosphorylation of LHCII (light harvesting complexes of photosystem II). The LTR to these redox signals induces changes in the accumulation of plastid *psaA* transcripts, in chlorophyll (Chl) fluorescence values *F*_\rm s_/*F*_\rm m_, in Chl *a/b* ratios and in transient starch accumulation in C3 plants. Maize seedlings grown in this light system exhibited a pronounced ability to perform both short-term and long-term acclimation at the level of *psaA* transcripts, Chl fluorescence values *F*_\rm s_/*F*_\rm m_ and Chl a/b ratios. Interestingly, maize seedlings did not exhibit redox-controlled variations of starch accumulation probably because of its specific differences in energy metabolism. In summary, the artificial laboratory light system was found to be well-suited to mimic field light conditions and provides a physiological tool for studying the molecular regulation of the LTR of maize in more detail.

## INTRODUCTION

Plants need to cope with natural variations in illumination and temperature during their vegetation period. The major process affected by both parameters is photosynthesis, which is both light- and temperature-sensitive due to the tight coupling between light and dark reaction (Hüner et al., 1998; Blankenship, 2002). Photosynthesis, therefore, represents an attractive target for biotechnological improvements in order to promote growth and yield of crop plants (Long et al., 2006; Zhu et al., 2010).

Natural illumination of plants is highly fluctuating in intensity and quality mainly because of seasonal and daily periodicity and of short-term disturbances caused from, e.g., clouding or leaf movement. Beside these abiotic influences plants themselves create strong light gradients especially within dense plant populations where leaves of neighboring plants shade each other and compete for light. This competition for light results in strong decreases in light intensity and a relative enrichment in far-red wavelengths within the canopy (Terashima et al., 2005). The latter effect is sensed by photoreceptors which control photomorphogenesis or shade avoidance responses (Ballare, 1999; Smith, 2000). The enrichment in far-red light wavelengths has an additional strong impact on photosynthesis since it can be partly used by photosystem I (PSI) but not or less effectively by photosystem II (PSII) causing imbalanced distribution of excitation energy between the photosystems. During evolution plants developed a number of responses which optimize light utilization under these variable conditions (Aro and Andersson, 2001; Blankenship, 2002). Developmental responses at the whole-plant or leaf level include variation of photosynthetic capacity by modification of leaf thickness (e.g., sun and shade leaves) which take place within weeks and months. They act in concert with more dynamic molecular acclimation mechanisms at the chloroplast level which work within the range of minutes to several hours. These physiological responses are fully reversible. In the short-term functionality of the photosystem antennae is adjusted by important regulatory processes such as non-photochemical quenching for pH dependent dissipation of excess excitation energy under high light as well as state transitions – a posttranslational modification, which counteracts imbalanced excitation of the photosystems under low light. In the long-term, changes in gene expression control an adjustment of photosystem stoichiometry which work in the same functional direction as state transitions, but create a longer lasting effect (Melis, 1991; Anderson et al., 1995; Allen and Pfannschmidt, 2000; Haldrup et al., 2001; Allen, 2003; Holt et al., 2004; Pons and de Jong-Van Berkel, 2004; Kanervo et al., 2005; Walters, 2005; Eberhard et al., 2008; Horton et al., 2008; Kargul and Barber, 2008). These different responses generate a hierarchical framework which countervails the high variability in illumination and optimize photosynthetic electron transport (Frenkel et al., 2007; Dietzel et al., 2008). Therefore, it was proposed that manipulation of photosynthetic acclimation might help to improve photosynthesis (Horton et al., 2001; Murchie et al., 2009). In general, photosynthetic acclimation appears to be a chloroplast-autonomous regulation being independent of photoreceptors (Walters et al., 1999; Fey et al., 2005a); however, because of the numerous interactions in the plant cellular signaling network indirect photoreceptor and hormone influences must be also taken into account (Boonman et al., 2007, 2009).

Photosystem stoichiometry adjustment could be found in shade- and sun- as well as in mono- and dicot plants (Chow et al., 1988, 1990a; Pfannschmidt et al., 1999a; Fan et al., 2007) and appears to be an ubiquitous acclimation response in photosynthetic organisms (Melis, 1991; Allen and Pfannschmidt, 2000). Recent studies with the model organism *Arabidopsis thaliana* have revealed that the thylakoid-associated kinase STN7 is a key regulator of this long-term response (LTR; Bonardi et al., 2005; Pesaresi et al., 2009). Using knock out mutants as negative control it could be demonstrated that state transitions and LTR provide a physiological advantage under permanently changing conditions resulting in enhanced growth and seed production (Bellafiore et al., 2005; Frenkel et al., 2007; Wagner et al., 2008). Furthermore, systems biology approaches revealed that photosynthetic acclimation responses not only reconfigure the photosynthetic apparatus to the instantaneous light environment but also trigger a metabolic reprogramming which coordinates the energetic demands with the light harvesting efficiencies of the plant (Bräutigam et al., 2009; Frenkel et al., 2009). The studies demonstrated that starch accumulation in *Arabidopsis* significantly differs between different light quality acclimation states and, thus, represents a well-suited reporter for the metabolic reprogramming that finally controls plant growth efficiency (Bräutigam et al., 2009).

Maize is one of the world’s most important field crops and improvement of its photosynthetic yield would be of great interest (Zhu et al., 2010). Maize fields generate tall and dense plant stands during its vegetation period, which contains strong light gradients and, hence, a high degree of photosynthetic acclimation should be expected. Recent observations demonstrated that maize exhibit a high ability for photosynthetic acclimation when grown under different white light (WL) intensities in growth chambers. Variations in antenna structures and LHC protein accumulation were found both in mesophyll and bundle sheath chloroplasts (Drozak and Romanowska, 2006).

Here, we analyzed the degree of photosynthetic acclimation in maize grown under low-intensity artificial light qualities, which preferentially excite either PSI or PSII. This test system was successfully used previously for understanding redox-controlled photosynthetic acclimation in C3 plants like mustard, tobacco and *Arabidopsis* (Pfannschmidt et al., 1999a, b, 2001; Fey et al., 2005b; Wagner et al., 2008). Light quality acclimation to artificial light quality gradients could be demonstrated to be efficient and beneficial in various C3 species (Chow et al., 1990b; Hogewoning et al., 2012). In order to test the corresponding response of the C4 plant we determined chlorophyll (Chl) a fluorescence, Chl a/b ratios, transcript accumulation of plastid gene *psaA* (encoding the core protein of PSI), phosphorylation state of light harvesting complexes of PSII (LHCII) and the ability to perform state transitions. The detected acclimation ability was compared to that of samples harvested from maize plants grown under natural conditions. Our study indicates a high ability of maize to acclimate to light quality gradients in low-intensity light conditions in a lab based system providing a tool for analyzing the potential of the maize LTR for biotechnology applications.

## RESULTS

### LIGHT GRADIENTS AND PHOTOSYNTHETIC ACCLIMATION OF MAIZE UNDER FIELD CONDITIONS

**FIGURE 1:**
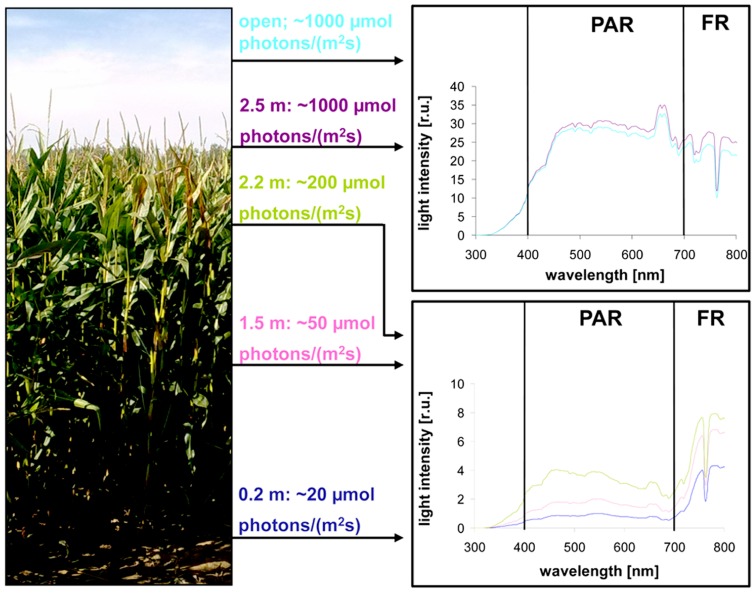
**Natural illumination conditions within the maize field used for sampling.** Left picture displays the plant stand used for measurements. Light intensities determined at indicated heights within the stand are given in the middle panel. Corresponding light spectra (indicated by the same color) at the respective position are shown on the right. Open: intensity and spectrum of full sunlight outside the field. PAR: photosynthetic active radiation. FR: far-red region.

**FIGURE 2:**
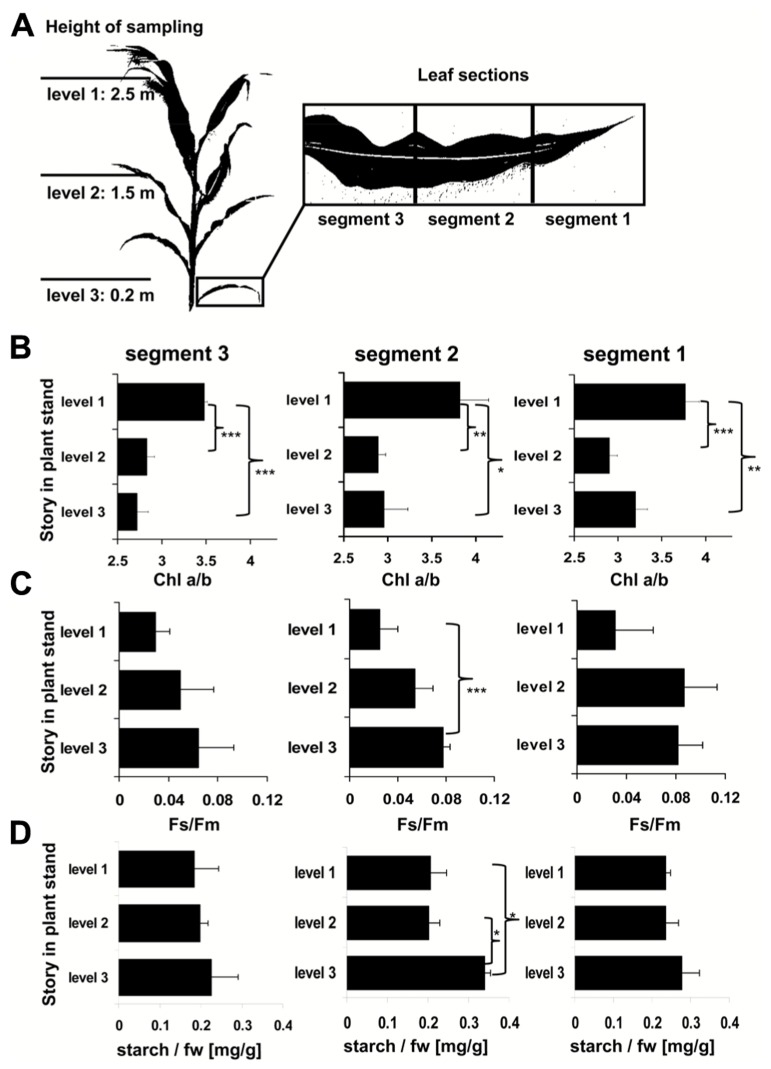
**Analysis of field grown maize. (A)** Left panel displays height of leaf harvesting. Magnification on right defines the analyzed sections of the respective leaves. Three to five leaves of each level were harvested from different individuals distributed randomly over the field. **(B)** Chl *a/b *ratio, **(C)** Chl fluorescence, and **(D)** starch contents in field grown maize. Story of harvest is given on the left, respective leaf segment analyzed on top. Acclimation parameters Chl *a/b* and *F*_s_/*F*_m_ were determined as described in Section “Materials and Methods.” Starch content is given relative to the fresh weight (fw). Data represent means from at least three independent biological replicates, standard deviation is given. A Student’s *t*-test was performed to test significance of differences (*P < 0.05, **P < 0.01, ***P < 0.005).

Light intensity within plant populations decreases as a function of the leaf area index (LAI) while at the same time an enrichment of far-red wavelengths occurs (Smith, 2000; Hirose, 2005). We wanted to test if we could use a laboratory light set-up to study acclimation responses of maize seedlings to natural light gradients. In order to have a reference we analyzed the course of light gradients in a maize field trail by determination of light intensity and spectrum at different heights (**Figure 1**) of full-grown maize plants. 80% of the light was absorbed within the top 10–20% of the field, all lower parts obtained low light intensities with a strong enrichment in the far-red range (**Table 1**). Thus, even under sunny conditions the majority of the leaves in the field perceived only a sub-saturating photosynthetic active radiation (PAR) with a three- to four-times higher proportion of far-red light. To analyze effects on the photosynthetic performance of the plants we harvested plant leaf samples from the top, the middle and the bottom region of the stand. To include possible effects of the leaf blade position and the developmental gradient in chloroplast biogenesis we studied three segments of the leaves (basal, middle, and top segment; **Figure 2A**). In order to study the potential light acclimation we probed the Chl *a/b* ratio as indicator for changes in the amount of LHCII and the Chl fluorescence value *F*_s_/*F*_m_ as physiological marker for the LTR, This value represents the fraction of light which is absorbed by the PSII antenna and subsequently lost by constitutive dissipation *via* fluorescence and heat emission in the steady state. Photosystem stoichiometry adjustment creates significant changes in this parameter (Pfannschmidt et al., 2001). The Chl *a/b* ratio was found to be significantly higher in level 1 than in level 2 and 3 (**Figure 2B**) while the measured *F*_s_/*F*_m_ values were lower in level 1 and increased step-wise toward level 2 and 3 (**Figure 2C**) with a significant difference in section 2 of level 1 and 3. Effects of the leaf gradient were hardly detectable with the exception of segment 1 in level 2 which displayed a slightly more pronounced increase in the *F*_s_/*F*_m_ values than segments 2 and 3. The starch content was found to be relatively stable at the different levels of sampling (**Figure 2D**) with a weak tendency to increase toward the bottom level which, however, was only significant in segment 2 of level 3. In summary, the measured parameters *F*_s_/*F*_m_ and Chl *a/b* demonstrated typical changes between top and bottom level being indicative for a functional LTR in maize under field conditions.

**Table 1:** Ratio of short and long wavelengths in the different field stories.

Position in field	R/FR
Outside	1.413 ± 0.002
2.5 m	1.351 ± 0.012
2.2 m	0.483 ± 0.068
1.5 m	0.561 ± 0.147
0.2 m	0.400 ± 0.070

*The ratio has been calculated from integration of the values measured in the wavelength ranges 655–665 (R: red) and 725–735 (FR: far-red) nm. Results represent means of three measurements, standard deviation is given.*

### PHOTOSYNTHETIC ACCLIMATION OF MAIZE SEEDLINGS TO LIGHT QUALITY GRADIENTS

**FIGURE 3:**
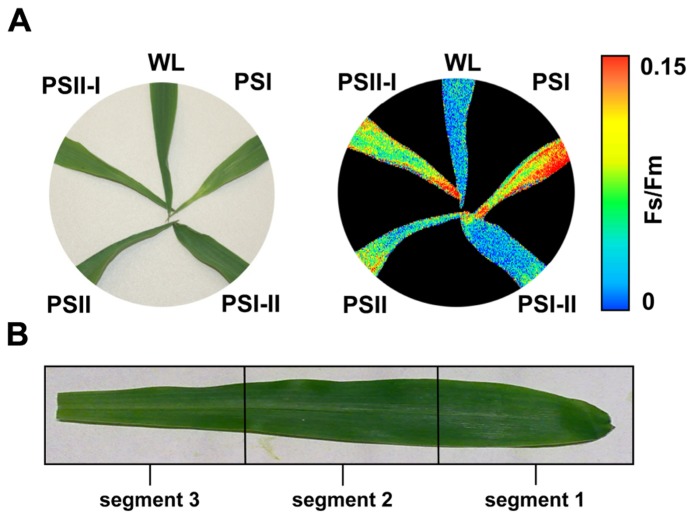
**Chl fluorescence of maize seedlings acclimated in long-term to different artificial light regimes affecting the redox state of photosynthetic electron transport.** The growth light regime is indicated next to each leaf. **(A)** Left: appearance of leaves of differently acclimated plants under white light. Right: *F*_s_/*F*_m_ values of the same leaves as detected by a CCD camera for Chl fluorescence detection given in false colors. Color bar on the right indicates the measured values. First and second true leaves were used for all analyses, the figures display representative samples. **(B)** Leaf sections used for more detailed analyses (compare **Figures 4 and 5**) were selected according to the field sampling.

Recently, we described the physiological and molecular acclimation of the dicotyledonous C3 plants *A. thaliana,* *Nicotiana tabacum*, and *Sinapis alba* grown in a light system which preferentially excites either PSI or PSII (Pfannschmidt et al., 2001; Fey et al., 2005b; Wagner et al., 2008; Steiner et al., 2009). Here, we used the same set-up to analyze photosynthetic acclimation in the monocotyledonous C4 plant *Zea mays*. Seedlings were grown under WL as control and under PSI- or PSII-light (PSI, PSII) in order to test their ability to perform a LTR. Additional controls were shifted between the PSI- and PSII-lights (PSI-II; PSII-I) to test the reversibility of the response, an important criterion to distinguish between acclimation and development. The general appearance of the plants grown under the different light regimes did not reveal major morphological differences which is consistent with earlier observations in C3 plants (**Figure 3A**). For detection of a LTR the second true leaves of 10-day-old plants were used for measuring the *F*_s_/*F*_m_ parameter by video imaging (**Figure 3A**). In this experimental set-up PSI-light acclimated plants absorb more light than they can use and dissipate the excess as fluorescence which usually is significantly higher in the steady-state than that from PSII-light acclimated plants. A deficiency in performing a LTR, however, is indicated by a loss of significant changes in *F*_s_/*F*_m_ as observed in the *stn7* mutant of *Arabidopsis* (Bonardi et al., 2005). WL plants exhibited a low *F*_s_/*F*_m_ value while PSI plants displayed a high one. After a shift from PSI-light to PSII-light the *F*_s_/*F*_m_ value in the seedlings declined. In PSII-plants it was low and increased after a shift of the plants to PSI-light. This result is consistent with earlier measurements of *F*_s_/*F*_m_ values in C3 plants indicating that maize seedlings are able to perform a LTR in the same manner.

**FIGURE 4:**
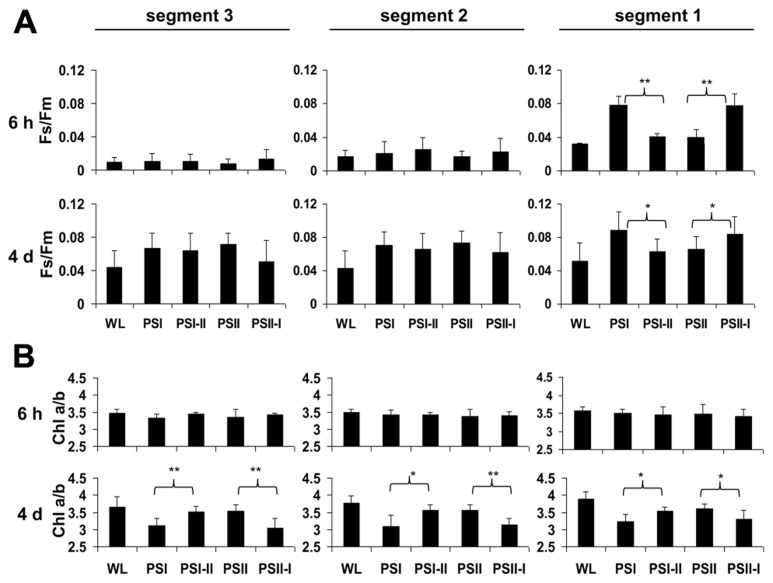
**Chl fluorescence and Chl *a/b* ratios in defined segments of maize seedlings acclimated in long-term to different artificial light qualities.** Growth light conditions are given below the charts. Plants under PSI-light were shifted to PSII-light and *vice versa *and harvested 6 h of 4 days after the shift (indicated in the left margin) and compared to non-shifted controls. **(A)** *F*_s_/*F*_m_ and **(B)** Chl *a/b* were determined from the same segments (indicated on top, compare Figure3). Data represent at least three independent biological replicates, standard deviation is given. A Student’s *t*-test was performed to test significance of differences (*P < 0.05, **P < 0.01).

2D images of the Chl fluorescence revealed some variations in the fluorescence values between different areas of the leaf blade (**Figure 3A**) which could be caused by the characteristic developmental gradient of the plastids in young monocotyledonous leaves which is more pronounced than in fully developed leaves as investigated in **Figure 2**. To examine this in more detail leaf blades were dissected into three segments containing young plastid from the leaf base (segment 3), middle aged plastids (segment 2), and mature plastids from the leaf tip (segment 1; **Figure 3B**). The *F*_s_/*F*_m_ values were determined 6 h and 4 days after either a PSI-II or a PSII-I light shift (**Figure 4A**) in comparison to the respective controls. 6 h after a light shift only segment 1 revealed significant changes in *F*_s_/*F*_m_ values. Segment 2 and 3 exhibited very weak Chl fluorescence without any recognizable differences between the growth light regimes. Four days after the light shift the Chl fluorescence in general was increased indicating the progress in the build-up of the photosynthetic machinery during the time range of observation. However, again segment 1 was the only segment displaying significant *F*_s_/*F*_m_ fluorescence changes characteristically for a LTR.

**Figure A1:**
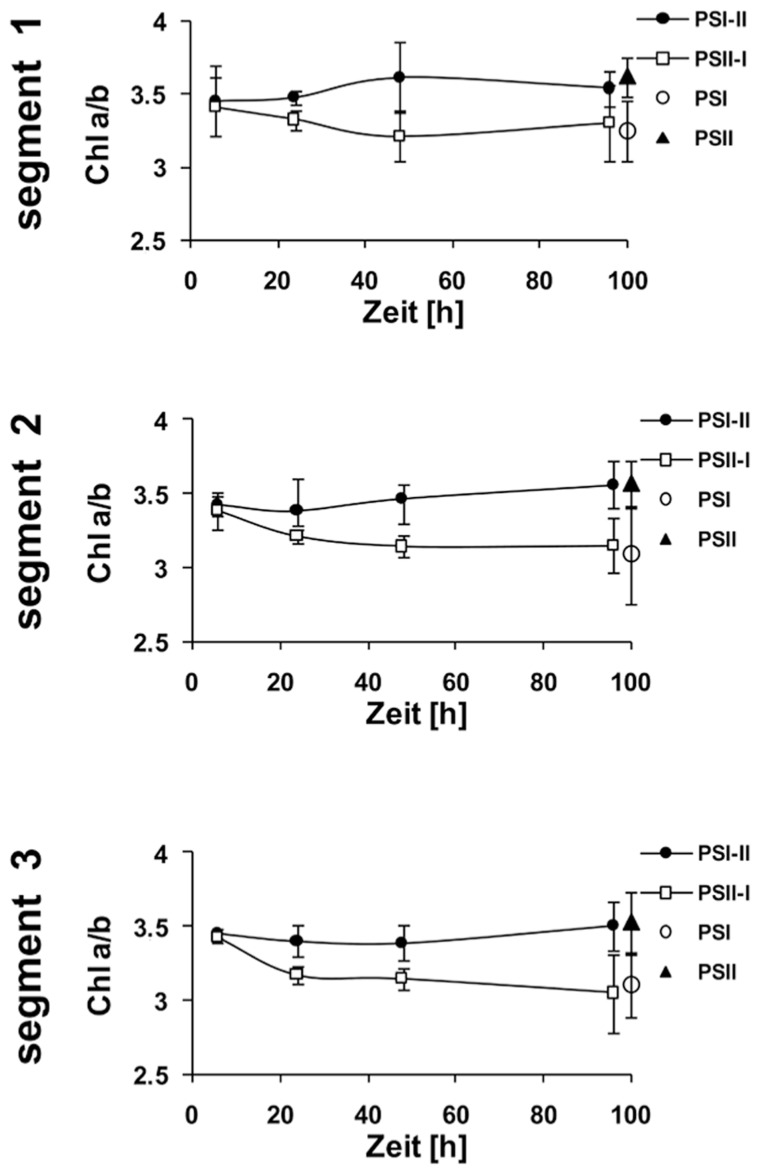
**Kinetic changes of Chi a/b ratio in defined segments of maize seedlings acclimated to different light qualities.** Growth light conditions are given in the right margins. Plants under PSI-light were shifted to PSII-light and *vice versa* and harvested at indicated time points after the shift and compared to non-shifted controls. Data represent at least three independent biological replicates, standard deviation is given.

In the same experiments we analyzed the Chl *a/b* ratios of the respective samples (**Figure 4B**) which represents a second reporter for the LTR. After 6 h of acclimation it was found to be stable and comparable to the controls. After 4 days, however, the Chl *a/b* ratio of the PSI-II plants was significantly higher than that of PSI plants resembling that of PSII and WL plants. In contrast, PSII-I plants exhibited the opposite behavior with a lower Chl *a/b* ratio as in PSI plants. This pattern was found in all three segments analyzed indicating that even the younger leaf segments had begun to acclimate to the light condition although the Chl fluorescence measurements yet did not indicate this. A more detailed time course experiment indicated that the changes in Chl *a/b* ratios were largely finished after 2 days of acclimation (**Figure A1** in Appendix) which is similar to the dynamics observed earlier in *Arabidopsis* (Wagner et al., 2008).

### REDOX-CONTROLLED MOLECULAR PROCESSES IN PHOTOSYNTHETIC ACCLIMATION

**FIGURE 5:**
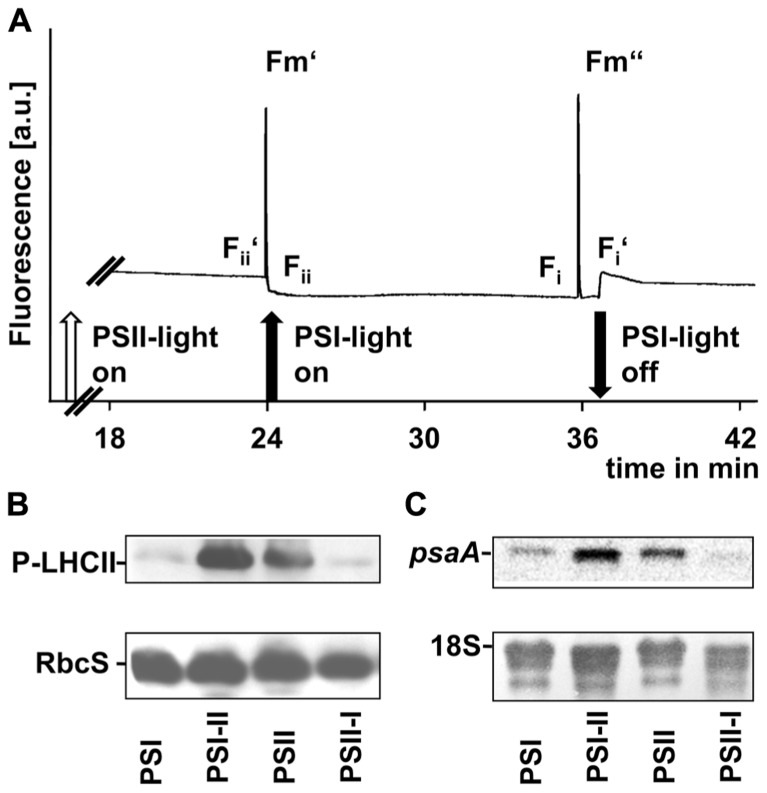
**Redox-controlled processes in photosynthetic acclimation of maize seedlings. (A)** Artificial growth lights were used as actinic light sources in state transition experiments to indicate the short-term redox effects on linear electron transport. Chl fluorescence given in arbitrary units was recorded using a PAM fluorometer. Measurements were done three times with leaves from three different biological samples. A typical trace is shown. On–off switches of the lights are given below the trace. Nomenclature of fluorescence parameters follows established conventions (see Materials and Methods). **(B)** Plastid protein extracts of maize seedlings grown under the different long-term light quality regimes as indicated in Section “Material and Methods” (indicated on bottom, compare **Figures 3 and 4**) were used to analyze the phosphorylation state of LHCII using a anti-phosphothreonine antibody. As loading control a corresponding experiment with the same membrane targeted to the small subunit of RubisCO (RbcS) is shown. **(C)** RNA isolates from the same samples isolated in parallel were used to determine the accumulation of *psaA* transcripts by northern analysis. As loading control a hybridization of the same membrane with a probe directed against the 18S rRNA is shown.

Shifts between the PSI- and PSII-lights (and *vice versa*) induce reduction or oxidation of the plastoquinone pool (Wagner et al., 2008), respectively, which serves as a sensor for the balanced action of the two photosystems. In C3 plants, it controls the transcriptional regulation of the plastid gene *psaA*, targets the phosphorylation of the LHCII and the performance of state transitions. We tested all three processes in segment 1 of the leaf blades (**Figure 5**).

To demonstrate the effect of light sources on the redox state of the electron transport chain we used actinic lights in standard state transitions measurements (Haldrup et al., 2001). Chl fluorescence was detected using a pulse amplitude modulation (PAM) fluorometer (**Figure 5A**). The Chl fluorescence traces displayed changes characteristic for state transitions as observed earlier in C3 plants, i.e., a slow transient rise after a sudden drop when the far-red light was switched on and a more rapid transient decrease after a sharp rise when it was switched off again. These fluorescence transients are caused by the lateral migration of the LHCII between PSII and PSI and indicate that the time range necessary for state transitions in maize corresponded to that observed in *Arabidopsis *(Wagner et al., 2008).

Western analyses of thylakoid membrane phosphorylation state using an anti-phosphothreonine antibody (**Figure 5B**) indicated a strong phosphorylation of the LHCII proteins under PSII- and PSI-II light conditions while only weak phosphorylation signals could be detected in samples obtained from PSI- and PSII-I plants. This observation is consistent with the Chl fluorescence measurements and indicates the activity of a redox-sensitive kinase catalyzing state transitions *via* LHCII phosphorylation.

Northern analyses of total RNA preparations indicated a *psaA* transcript pool in PSI-light adapted plants which increased after a shift into PSII-light (**Figure 5C**). A similar transcript accumulation was observable in PSII-light grown plants and a down-regulation of *psaA* transcript amounts became apparent after a shift to PSI-light (**Figure 5C**).

In total all three parameters clearly demonstrated that redox-controlled acclimation responses occur in maize and that the seedlings perform a proper LTR at least in segment 1 under the artificial light quality regimes.

**FIGURE 6:**
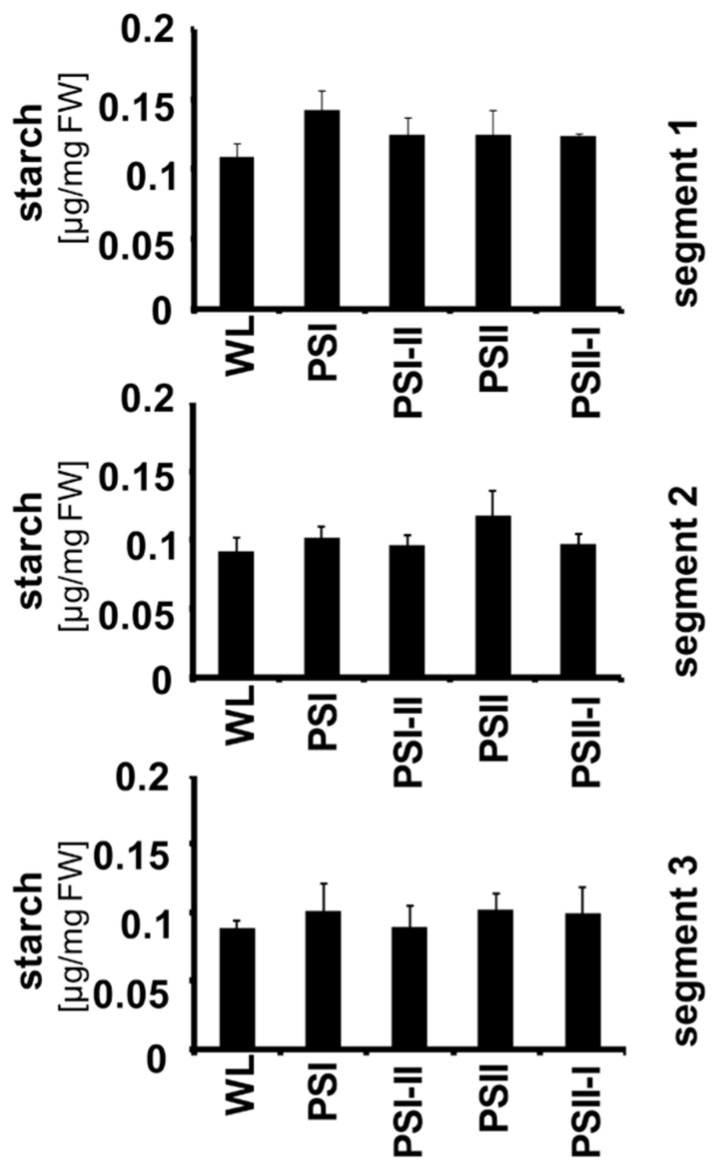
**Starch accumulation in segments of maize seedlings grown in long-term under the different light quality regimes.** Starch per fresh weight (FW) was determined from the three leaf segments (indicated on the right) as described before. Growth light regimes of the respective plant samples are indicated at the bottom of each panel. All determinations were done in triplicate, standard deviations are given.

Recently, it could be demonstrated that the LTR includes targeted changes in the metabolic state of *Arabidopsis* and that this is reflected by a characteristic increase in the starch content of the leaves under PSII and PSI-II lights when compared to PSI and PSII-I conditions. The *stn7* mutant was unable to perform this increase indicating that it is an redox-controlled process (Bräutigam et al., 2009). To test whether this regulation is also present in maize, seedlings were grown under the four light regimes and starch accumulation was determined in each segment (**Figure 6**). In contrast to *Arabidopsis* we could not observe any distinct pattern of starch accumulation in response to the light quality indicating that this regulation does not work in maize although the photosynthetic acclimation processes appeared to be fully functional. This result is consistent with the findings in plants collected from the field.

### REDOX-DEPENDENT PHOSPHORYLATION OF LHCII IN MAIZE GROWN IN THE FIELD OR UNDER VARIOUS LABORATORY CONDITIONS

**FIGURE 7:**
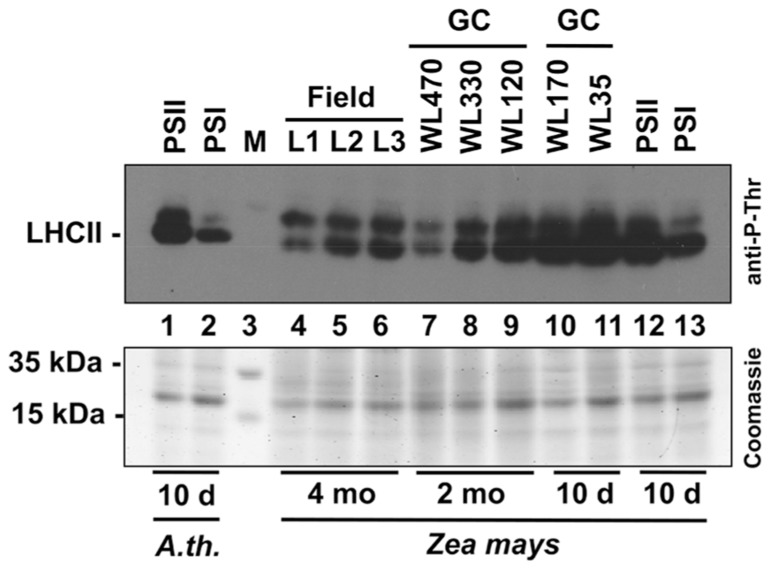
**Redox-dependent phosphorylation of LHCII in maize grown under field conditions or various laboratory conditions.** Protein extracts of differentially grown plants were separated on a denaturing 10% SDS-PAGE, blotted to a PVDF membrane and subjected to an immunoblot analysis using an anti-phosphothreonine antibody (top panel). Signals were visualized by enhanced chemiluminescence. 20 μg total protein were loaded per lane. A parallel control gel was loaded with the same protein amounts from the same samples and subjected to Coomassie staining as loading control (bottom panel). Lanes 1 and 2: extracts of 10-day-old *Arabidopsis* seedlings grown under artificial PSII- and PSI-light serving as control. Lane 3: size marker. Lanes 4–6: Leaf protein extracts of 4 months old field grown maize (L1–L3 correspond to the respective height levels given in **Figures 1 and 2**). Lanes 7–9: leaf protein extracts of 2 months old maize plants grown in a growth chamber (GC) equipped with mercury vapor white light sources. Light intensity at the height of leaf harvest is given in μmol photons per m^2^ and s. Lanes 10 and 11: leaf protein extracts from 10-day-old maize plants grown in growth cabinets equipped with cool white light fluorescent stripe lamps (light intensity is given in μmol photons per m^2^ and s). Lanes 12 and 13: leaf protein extracts of 10-day-old maize plants grown under the same PSII- and PSI-light conditions as given for extracts from *Arabidopsis *seedlings presented in lanes 1 and 2. All plants used for experiments given in lanes 7–13 were grown in the same growth chamber being subjected to 60% relative humidity and 18–21°C.

In order to obtain a direct comparison of acclimation abilities of maize under field and laboratory conditions we determined the phosphorylation state of the LHCII using immunoblotting analyses with antibodies directed against phosphothreonine. We harvested leaf material in the field corresponding to the levels 1–3 (compare **Figure 2**) and isolated total leaf protein extracts. 20 μg protein were separated by SDS gel electrophoresis and subjected to immunoblot analysis (**Figure 7**). In parallel, we separated the same amounts of protein extracts isolated from 2 months old maize plants grown in growth chambers where they experienced mainly a light intensity gradient due to vertical growth without neighboring plants. In addition, we grew maize seedlings for 10 days directly under two different WL intensities (170 and 35 μE) as well as in our light quality regime. For the latter *Arabidopsis* plants grown in parallel were used as control. Redox-mediated LHCII phosphorylation induced by growth under PSII- and PSI-lights appeared to be comparable for *Arabidopsis* and maize seedlings (**Figure 7**, lanes 1, 2 and 12, 13) and resulted in a strong phosphorylation of LHCII under PSII-light and a weak phosphorylation under PSI-light, respectively. This result is in accordance with our earlier observations (Wagner et al., 2008, **Figure 5**). Field grown maize exhibited a phosphorylation state of LHCII at levels 2 and 3 (lanes 5, 6) being comparable to that of the PSI-light control (lane 13) while that of level 1 (lane 4) was clearly reduced. The latter observation corresponds to reports demonstrating that the LHCII kinase is inhibited at high light intensities by a thiol-dependent repression mechanism (Rintamaki et al., 2000). Interestingly, the phosphorylation signal occurring above the LHCII remained stable under all conditions. Because of size and regulation behavior of this band under lab conditions it is likely the D1 protein which requires experimental proof in the future. If this assumption is correct then the stable phosphorylation contrasts results from laboratory experiments with *Arabidopsis* which suggest that D1 phosphorylation is affected by light intensity (Tikkanen et al., 2008; compare also lanes 7–9) pointing to potential additional control mechanisms in field grown maize besides light-dependent redox control. Maize from growth chambers not subjected to dense planting displayed a regular increase in LHCII phosphorylation from the top level to the bottom (lanes 7–9) which can be easily explained by the light-intensity dependent repression of the kinases. The additional experiment with the 10-day-old maize seedlings grown under 170 and 35 μE WL (lanes 10 and 11) displaying high phosphorylation states under both conditions indicates that a significant high light repression of LHCII phosphorylation in maize likely starts beyond the 330 μE light intensity point (Lane 8). This is much higher as the corresponding point in *Arabidopsis* (about 150 μE, compare Bonardi et al., 2005), probably since maize is genotypically adapted to much higher light intensities for growth than *Arabidopsis*. In summary, we conclude that the principal LHCII phosphorylation events occurring in the field can be mimicked by our artificial light quality system. Phosphorylation of the putative D1 protein, however, appears to be potentially under a more complex control including additional influences besides the light environment and requires more complex physiological set-ups in order to study it. 

## DISCUSSION

Maize is one of the major crops in worldwide agriculture and, therefore, its ability to respond and acclimate to changing environmental conditions is of great interest, especially with regard to rising CO_2_ concentrations in the atmosphere (Prins et al., 2007). Recent analyses have demonstrated that the acclimation to CO_2_ is closely related to the water status and the developmental stage of the leaf investigated reflecting the multiple effects on photosynthesis (Prins et al., 2011). The efficiency of CO_2_ fixation, however, also largely depends on the energy provided by the light reaction which might create a limiting factor since it is well-known that maize fields possess a very high LAI. Our measurements of the light gradients within such a field confirm this and demonstrate that in a fully grown maize field approximately 70–80% of the biomass can perform photosynthesis only in a very sub-optimal way since the useful light wavelengths as driving force for the light reaction are very limited. This occurs even under optimal sun light conditions suggesting that there is a high potential for improvement of light usage efficiency in the same way as discussed for rice (Horton et al., 2001).

Recently, molecular studies investigated the molecular changes of the photosynthetic apparatus in maize in response to WL intensity gradients (Romanowska and Drozak, 2006; Romanowska et al., 2008). In the present study we focused on potential changes in photosynthetic functions in maize caused by long-term variations of incident light quality using artificial light sources exciting preferentially PSI or PSII. A similar approach has been performed earlier, however, with the main focus on the effectiveness of the phytochrome system (Eskins et al., 1986). A recent study explains photosynthetic acclimation to canopy density of tobacco and *Arabidopsis* as an interplay of photoreceptors and cytokinins, but the results also suggest a possible existence of alternative signaling pathways (Boonman et al., 2009). Our physiological set-up was originally established to analyze processes controlled by redox signals from photosynthetic electron transport in C3 plants. Our experiments presented here demonstrate that the system is able to induce a prominent short-term response also in the maize seedlings. Redox-controlled state transitions as measured by Chl fluorescence and corresponding changes in LHCII phosphorylation (**Figure 5**) were observable in a comparable way as reported earlier (Andrews et al., 1993). In addition, we could detect a pronounced LTR with corresponding changes in *psaA* transcript accumulation (**Figure 5C**) and characteristic changes in *F*_s_/*F*_m_ and Chl *a/b* values (**Figure 4**). The LTR, however, was fully observable only in the top leaf segment of the maize seedlings indicating a developmental dependency of the LTR on the gradient of chloroplast biogenesis at least under the conditions of the present study (**Figure 4**). In contrast, in mature leaves of field grown plants the LTR as reported by respective changes of *F*_s_/*F*_m_ and Chl *a/b* was readily detectable in all segments (**Figure 2**) supporting this conclusion. Our light measurements in the field confirm earlier results demonstrating that in dense plant population strong light quality gradients exist which are present even under sunny conditions (**Figure 1**). It can be concluded that fluctuations of illumination from outside are dampened in their absolute values by the top layers of the field and that within the field canopy the light environment is a rather stable low-intensity, far-red enriched light condition. Thus, leaves in the top level mainly require to perform high light acclimation using mechanisms such as non-photochemical quenching (NPQ). In contrast, for the largest portion of the biomass in the field a long-term acclimation to the non-saturating low light conditions appears to be more desirable. We could observe a distinct difference between the *F*_s_/*F*_m_ and Chl *a/b* values for the top storey of the field when compared to middle or bottom storey indicating an adjustment of the photosynthetic apparatus to the respective illumination conditions (**Figure 2**). Leaf autonomous regulation of photosynthetic acclimation *via* redox signals from photosynthesis would provide an efficient and simple regulation principle allowing the individual plants to adapt to the strong gradient in light intensity on one hand and the parallel light quality gradient on the other. Thus, our physiological light system and the reporter for the LTR provide a useful test system not only for redox-controlled processes, but also for mimicking field conditions in dense canopies.

In *Arabidopsis *we observed a concomitant variation of the starch amount during the LTR which was reported to be a reporter for the metabolic changes occurring in the background of the light acclimation response (Bräutigam et al., 2009). Interestingly, this was not observable in maize, neither in the seedlings nor in the mature plants. In *Arabidopsis,* it was shown that starch synthesis is dependent on redox regulation of the entry enzyme ADP-glucose-pyrophosphorylase (AGPase) *via* the thioredoxin-like NtrC (Michalska et al., 2009). It is not clear yet whether the changes in starch accumulation during the LTR are also mediated by NtrC, however, it is a likely candidate. In maize obviously the regulatory link between LTR and starch accumulation has been uncoupled. A possible explanation would be the C4 syndrome of maize, since in C4 metabolism starch is mainly produced in the bundle sheath cells which do not contribute significantly to overall linear electron transport, thus lacking the basic requirement for photosynthetic redox signaling. However, it could be shown that even the agranal chloroplasts of the bundle sheath cells of maize contain a certain amount of PSII providing at least a limited capacity for redox signaling (Romanowska et al., 2008). BLAST searches done with the NtrC protein sequence of *Arabidopsis* identify an ortholog in maize with 92% positive amino acids strongly suggesting the existence of this key redox regulator in maize. Interestingly, starch accumulation in bottom leaves of the field grown plants is comparable or even slightly higher than in the top level leaves. Thus, starch accumulation appears to be uncoupled from the major site of primary photosynthesis implying a number of questions concerning the source–sink relationships in this plant which are an interesting topic for future work.

Regarding the high proportion of the field biomass performing a LTR the question arises for the beneficial effects of this acclimation response. In *Arabidopsis,* a clear negative impact on rosette growth and seed production could be demonstrated when the LTR is lacking (Wagner et al., 2008; Bräutigam et al., 2009). A recent study analyzed in detail the wavelengths dependency of photosynthetic quantum yield indicating significant positive effects for plants when performing a corresponding acclimation response (Hogewoning et al., 2012). Comparable experiments in maize require the isolation of corresponding LTR mutants which are currently under way. In addition, further field experiments are required to understand temporal and spatial occurrence of the LTR during the development of the plant stand. Young and small maize seedlings in the field perceive much more high light per total leaf area than the older plants which need to compete for light. Thus, the requirement for a LTR in the lower parts of the plants increases with the development of the plant stand. Understanding the molecular regulation of these processes will help to assess the potential of yield improvement in crop field *via* engineering of photosynthetic acclimation responses.

## MATERIALS AND METHODS

### FIELD GROWN PLANT MATERIAL

For field analyses we characterized a plot in the middle of a fully grown maize field near Jena on the 9th of September 2009 at a time point when development of the corncobs has not yet started. The density of plants within the field was between 16 and 20 plants/m^2^. Light conditions in the maize field were measured at different indicated heights at noon under sunny and dry conditions and 22°C air temperature. Light intensity of PAR was determined with a Licor LI-250 (Heinz Walz GmbH, Effeltrich, Germany). Light spectra were determined with a spectroradiometer GER 1.500 (Geophysical and Environmental Research Corp., Millbrook, NY, USA). Reference data were obtained outside the field in a plant free area nearby which was also free of reflecting light from the fields around. Based on these measurements leaves were harvested at three different heights with characteristic light conditions. Only healthy, green leaves without any sign of beginning senescence or pathogen attack were chosen. Leaves were put in a light-proof box on ice and transported immediately to the lab. Chl fluorescence measurements were started within 30 min after harvest and after acclimation to room temperature. Material for Chl and starch determination was frozen in liquid nitrogen and kept at -80°C until further use.

### PLANT MATERIAL GROWN IN CLIMATE CHAMBERS

For analysis of young maize seedlings *Z. mays* (L.) var seeds were sown in pots on soil/vermiculite (4:1) and grown in growth chambers at 25°C, 80% humidity and a 16 h light/8 h dark cycle. Illumination was ~30 μmol photons/m^2^s provided by cool white fluorescence lamps. Seedlings were grown for 3–4 days until the first leaf had developed. Then pots were shifted for further 3 days to light cabinets equipped with light sources preferentially exciting PSI or PSII (PSI- or PSII-light, respectively). The spectral composition of the light sources have been described in detail earlier (Wagner et al., 2008). Both light sources (PSI- and PSII-light) had an comparable PAR of 20–30 μmol photons/m^2^s which was found to be sufficient to sustain photoautotrophic growth. After this pre-acclimation one half of the plants were shifted from PSII- to PSI-light (PSII-I plants) and from PSI- to PSII-light (PSI-II plants) while the other half remained in the light cabinets (PSI and PSII plants). Plants were then re-acclimated for further 4 days before they were harvested for analyses. In time course experiments samples were taken 6, 24, 48, and 96 h after the light shift. Maize plants grown under high WL conditions or for prolonged growth under WL were subjected to illumination from mercury vapor WL sources in the same growth chambers as the PSI- and PSII-light sources. Light intensities as indicated in the figures were obtained by adjusting the distance of the plants to the light sources placed on top of them. The respective PAR intensity was detected using the Licor LI-250 (see above).

### CHLOROPHYLL FLUORESCENCE MEASUREMENTS

*In vivo* Chl *a* fluorescence parameters were determined by video imaging under room temperature using a pulse amplitude-modulated FluorCam 700 MF device (Photon Systems Instruments, Brno, Czech Republic). *F*_s_/*F*_m_ and *F*_v_/*F*_m_ of plants acclimated to different light qualities were determined as described earlier (Wagner et al., 2008). We also compared basic photosynthetic parameters such as *F*_v_/*F*_m_ detected by the Fluorcam with that of a PAM2000 (Heinz Walz GmbH, Effeltrich, Germany) and found the saturation flash of approximately 2000 μE sufficient to close all PSII centers for determination of *F*_m_. State transitions****were determined as described earlier (Lunde et al., 2000; Damkjaer et al., 2009) with intact single plant leaves using a PAM-101 fluorometer equipped with a PDA100 for digital data recording (WALZ, Effeltrich, Germany). PSII- and PSI-favoring light was provided by the growth light sources as described (Wagner et al., 2008). Saturation pulses were given for 800 ms by a Schott KL-1500 lamp at 5000 μmol photons/m^2^s. One representative of three replicates is shown.

### CHLOROPHYLL AND STARCH DETERMINATION

Plant material was ground in liquid nitrogen in a mortar. For determination of Chl concentration the pigments were extracted with 80% buffered acetone. Chl *a* and *b* concentrations were then spectroscopically determined according to Porra et al. (1989). Starch was determined using a standard colorimetric method (Magel, 1991) and the absorption coefficients described by Appenroth et al. (2010).

### NORTHERN ANALYSIS

Preparation of total RNA from the top section of 10-day-old maize seedlings grown in the light quality system as described above was performed as established earlier (Fey et al., 2005b). 10 μg of total RNA were separated on a 1% denaturing agarose gel containing 7% formaldehyde, transferred to a nylon membrane and hybridized to a probe directed against a highly conserved sequence in the *psaA* gene (encoding the apoprotein of PSI) of *Arabidopsis* following established standard protocols (Sambrook et al., 1989).

### PHOSPHORYLATION STATE OF LHCII

Fresh leaf tissue of differentially grown plants was homogenized using a Waring^®^ blender in ice cold homogenization buffer (HB; 50 mM HEPES-KOH, 0.33 M sorbitol, 10 mM KCl, 5 mM MgCl_2_, 10 mM NaF) at 4°C. Chloroplasts were recovered by centrifugation for 2 min at 2000 × *g.* Total chloroplast protein extracts containing 20 μg Chl were solubilized for 10 min in 40 μl buffer containing β-dodecyl-maltoside (BDM) at a final concentration of 1%. Proteins were denatured with 40 mM DTT, 2% SDS, 4% glycerol, and 150 mM Tris–HCL *pH* 6.8 for 10 min and subjected to SDS-PAGE using a 12% gel. The gel was either stained with colloidal Coomassie following manufacturer’s recommendations (Roth, Karlsruhe, Germany) or used for western analysis. The stained gel was scanned with a LI-COR Odyssey infrared laser scanner at 700 nm to detect the dye fluorescence and protein loading was determined by quantification of fluorescence signals. Calculations of relative protein amounts were done with LI-COR Odyssey2.1 software (median background correction). For immunoblot analysis gels were electro-transferred to a polyvinylidene difluoride (PVDF) membrane (Roti-PVDF, Roth, Karlsruhe, Germany) and de-stained according to current protocols (Wittig et al., 2006). Phosphorylated proteins were detected with a polyclonal primary anti-phosphothreonine antibody (Cell Signaling Technology Inc., Danvers, MA, USA) and a fluorophore-coupled secondary antibody IRD800-anti-rabbit (Rockland Immunochemicals Inc., Gilbertsville, PA, USA). For testing phosphorylation state of field material the harvested leaves were frozen on dry ice directly at the site of harvest to avoid any change in phosphorylation during transportation. Total leaf protein extracts were isolated according to Pfalz et al. (2009) and used for immune-western analysis with anti-phosphothreonine antibodies as described above with the exception that the detection was performed with enhanced chemiluminescence (ECL). Samples collected from lab growth chamber-grown plants were treated in an identical manner to allow direct comparison.

## Conflict of Interest Statement

The authors declare that the research was conducted in the absence of any commercial or financial relationships that could be construed as a potential conflict of interest.

## References

[B1] Allen J. F. (2003). State transitions – a question of balance. *Science* 299 1530–1532. 10.1126/science.1082833 12624254

[B2] Allen J. F. Pfannschmidt T. (2000). Balancing the two photosystems: photosynthetic electron transfer governs transcription of reaction centre genes in chloroplasts. *Philos. Trans. R. Soc. Lond. B Biol. Sci.* 355 1351–1357. 10.1098/rstb.2000.0697 11127990 PMC1692884

[B3] Anderson J. M. Chow W. S. Park Y.-I. (1995). The grand design of photosynthesis: acclimation of the photosynthetic apparatus to environmental cues. *Photosyn. Res.* 46 129–139. 10.1007/BF00020423 24301575

[B4] Andrews J. R. Bredenkamp G. J. Baker N. R. (1993). Evaluation of the role of state transitions in determining the efficiency of light utilisation for CO2 assimilation in leaves. *Photosyn. Res.* 38 15–26. 10.1007/BF00015057 24317826

[B5] Appenroth K. J. Krech K. Keresztes A. Fischer W. Koloczek H. (2010). Effects of nickel on the chloroplasts of the duckweeds *Spirodela polyrhiza* and *Lemna minor* and their possible use in biomonitoring and phytoremediation. *Chemosphere* 78 216–223. 10.1016/j.chemosphere.2009.11.007 19945735

[B6] Aro E.-M. Andersson B. (2001). *Regulation of Photosynthesis* Vol. 11 Dordrecht: Kluwer Academic Publishers.

[B7] Ballare C. L. (1999). Keeping up with the neighbours: phytochrome sensing and other signaling mechanisms. *Trends Plant Sci.* 4 97–102. 10.1016/S1360-1385(99)01383-7 10322540

[B8] Bellafiore S. Barneche F. Peltier G. Rochaix J. D. (2005). State transitions and light adaptation require chloroplast thylakoid protein kinase STN7. *Nature* 433 892–895. 10.1038/nature03286 15729347

[B9] Blankenship R. E. (2002).*Molecular Regulation of Photosynthesis*. Oxford: Blackwell Science Ltd 10.1002/9780470758472

[B10] Bonardi V. Pesaresi P. Becker T. Schleiff E. Wagner R. Pfannschmidt T. (2005). Photosystem II core phosphorylation and photosynthetic acclimation require two different protein kinases. *Nature* 437 1179–1182. 10.1038/nature04016 16237446

[B11] Boonman A. Prinsen E. Gilmer F. Schurr U. Peeters A. J. M. Voesenek L. A. C. J., et al. (2007). Cytokinin import rate as a signal for photosynthetic acclimation to canopy light gradients. *Plant Physiol.* 143 1841–1852. 10.1104/pp.106.094631 17277095 PMC1851814

[B12] Boonman A. Prinsen E. Voesenek L. A. C. J. Pons T. L. (2009). Redundant roles of photoreceptors and cytokinins in regulating photosynthetic acclimation to canopy density. *J. Exp. Bot.* 60 1179–1190. 10.1093/jxb/ern364 19240103 PMC2657547

[B13] Bräutigam K. Dietzel L. Kleine T. Ströher E. Wormuth D. Dietz K. J. (2009). Dynamic plastid redox signals integrate gene expression and metabolism to induce distinct metabolic states in photosynthetic acclimation in *Arabidopsis*. *Plant Cell* 21 2715–2732. 10.1105/tpc.108.062018 19737978 PMC2768923

[B14] Chow W. S. Anderson J. M. Hope A. B. (1988). Variable stoichiometries of photosystem II to photosystem I reaction centres. *Photosyn. Res.* 17 277–281. 10.1007/BF00035454 24429774

[B15] Chow W. S. Anderson J. M. Melis A. (1990a). The photosystem stoichiometry in thylakoids of some Australian shade-adapted plant-species. *Aust. J. Plant Physiol.* 17 665–674. 10.1071/PP9900665

[B16] Chow W. S. Melis A. Anderson J. M. (1990b). Adjustments of photosystem stoichiometry in chloroplasts improve the quantum efficiency of photosynthesis. *Proc. Natl. Acad. Sci. U.S.A.* 87 7502–7506. 10.1073/pnas.87.19.7502 11607105 PMC54775

[B17] Damkjaer J. T. Kereiche S. Johnson M. P. Kovacs L. Kiss A. Z. Boekema E. J. (2009). The photosystem II light-harvesting protein Lhcb3 affects the macrostructure of photosystem II and the rate of state transitions in *Arabidopsis*. *Plant Cell* 21 3245–3256. 10.1105/tpc.108.064006 19880802 PMC2782274

[B18] Dietzel L. Bräutigam K. Pfannschmidt T. (2008). Photosynthetic acclimation: state transitions and adjustment of photosystem stoichiometry – functional relationships between short-term and long-term light quality acclimation in plants. *FEBS J.* 275 1080–1088. 10.1111/j.1742-4658.2008.06264.x 18318835

[B19] Drozak A. Romanowska E. (2006). Acclimation of mesophyll and bundle sheath chloroplasts of maize to different irradiances during growth. *Biochim. Biophys*. Acta 1757 1539–1546. 10.1016/j.bbabio.2006.09.001 17034754

[B20] Eberhard S. Finazzi G. Wollman F. A. (2008). The dynamics of photosynthesis. *Annu. Rev. Genet.* 42 463–515. 10.1146/annurev.genet.42.110807.091452 18983262

[B21] Eskins K. Mccarthy S. A. Dybas L. Duysen M. (1986). corn chloroplast development in weak fluence rate red-light and in weak fluence rate red plus far-red light. *Physiol. Plant.* 67 242–246. 10.1111/j.1399-3054.1986.tb02450.x

[B22] Fan D. Y. Hope A. B. Smith P. J. Jia H. Pace R. J. Anderson J. M. (2007). The stoichiometry of the two photosystems in higher plants revisited. *Biochim. Biophys. Acta* 1767 1064–1072. 10.1016/j.bbabio.2007.06.001 17618597

[B23] Fey V. Wagner R. Brautigam K. Pfannschmidt T. (2005a). Photosynthetic redox control of nuclear gene expression. *J. Exp. Bot.* 56 1491–1498. 10.1093/jxb/eri180 15863445

[B24] Fey V. Wagner R. Brautigam K. Wirtz M. Hell R. Dietzmann A. (2005b). Retrograde plastid redox signals in the expression of nuclear genes for chloroplast proteins of *Arabidopsis thaliana*. *J. Biol. Chem.* 280 5318–5328. 10.1074/jbc.M406358200 15561727

[B25] Frenkel M. Bellafiore S. Rochaix J. D. Jansson S. (2007). Hierarchy amongst photosynthetic acclimation responses for plant fitness. *Physiol. Plant.* 129 455–459. 10.1111/j.1399-3054.2006.00831.x

[B26] Frenkel M. Kulheim C. Johansson Jankanpaa H. Skogstrom O. Dall’Osto L. Agren J. (2009). Improper excess light dissipation in *Arabidopsis* results in a metabolic reprogramming. *BMC Plant Biol.* 9:12 10.1186/1471-2229-9-12 PMC2656510 19171025

[B27] Haldrup A. Jensen P. E. Lunde C. Scheller H. V. (2001). Balance of power: a view of the mechanism of photosynthetic state transitions. *Trends Plant Sci.* 6 301–305. 10.1016/S1360-1385(01)01953-7 11435168

[B28] Hirose T. (2005). Development of the Monsi–Saeki theory on canopy structure and function. *Ann. Bot.* 95 483–494. 10.1093/aob/mci047 15585544 PMC4246794

[B29] Hogewoning S. W. Wientjes E. Douwstra P. Trouwborst G. van Ieperen W. Croce R. (2012). Photosynthetic quantum yield dynamics: from photosystems to leaves. *Plant Cell* 24 1921–1935. 10.1105/tpc.112.097972 22623496 PMC3442578

[B30] Holt N. E. Fleming G. R. Niyogi K. K. (2004). Toward an understanding of the mechanism of nonphotochemical quenching in green plants. *Biochemistry* 43 8281–8289. 10.1021/bi0494020 15222740

[B31] Horton P. Johnson M. P. Perez-Bueno M. L. Kiss A. Z. Ruban A. V. (2008). Photosynthetic acclimation: does the dynamic structure and macro-organisation of photosystem II in higher plant grana membranes regulate light harvesting states? *FEBS J.* 275 1069–1079. 10.1111/j.1742-4658.2008.06263.x 18318834

[B32] Horton P. Murchie E. H. Ruban A. V. Walters R. G. (2001). “Increasing rice photosynthesis by manipulation of the acclimation and adaptation to light,” in *Rice Biotechnology: Improving Yield, Stress Tolerance and Grain Quality* Vol. 236 ed. Novartis Foundation (Hoboken John Wiley & Sons) 117–134. 10.1002/9780470515778.ch9 11387974

[B33] Hüner N. P. A. Oquist G. Sarhan F. (1998). Energy balance and acclimation to light and cold. *Trends Plant Sci.* 3 224–230. 10.1016/S1360-1385(98)01248-5

[B34] Kanervo E. Suorsa M. Aro E. M. (2005). Functional flexibility and acclimation of the thylakoid membrane. *Photochem. Photobiol. Sci.* 4 1072–1080. 10.1039/b507866k 16307125

[B35] Kargul J. Barber J. (2008). Photosynthetic acclimation: structural reorganisation of light harvesting antenna – role of redox-dependent phosphorylation of major and minor chlorophyll a/b binding proteins. *FEBS J.* 275 1056–1068. 10.1111/j.1742-4658.2008.06262.x 18318833

[B36] Long S. P. Zhu X.-G. Naidu S. L. Ort D. R. (2006). Can improvement in photosynthesis increase crop yields? *Plant Cell Environ.* 29 315–330. 10.1111/j.1365-3040.2005.01493.x 17080588

[B37] Lunde C. Jensen P. E. Haldrup A. Knoetzel J. Scheller H. V. (2000). The PSI-H subunit of photosystem I is essential for state transitions in plant photosynthesis. *Nature* 408 613–615. 10.1038/35046121 11117752

[B38] Magel E. (1991). Qualitative and quantitative determination of starch by a colorimetric method. *Starch* 43 384–387. 10.1002/star.19910431003

[B39] Melis A. (1991). Dynamics of photosynthetic membrane composition and function. *Biochim. Biophys. Acta* 1058 87–106. 10.1016/S0005-2728(05)80225-7

[B40] Michalska J. Zauber H. Buchanan B. B. Cejudo F. J. Geigenberger P. (2009). NTRC links built-in thioredoxin to light and sucrose in regulating starch synthesis in chloroplasts and amyloplasts. *Proc. Natl. Acad. Sci. U.S.A.* 106 9908–9913. 10.1073/pnas.0903559106 19470473 PMC2700983

[B41] Murchie E. H. Pinto M. Horton P. (2009). Agriculture and the new challenges for photosynthesis research. *New Phytol.* 181 532–552. 10.1111/j.1469-8137.2008.02705.x 19140947

[B42] Pesaresi P. Hertle A. Pribil M. Kleine T. Wagner R. Strissel H. (2009). *Arabidopsis* STN7 kinase provides a link between short- and long-term photosynthetic acclimation. *Plant Cell* 21 2402–2423. 10.1105/tpc.108.064964 19706797 PMC2751956

[B43] Pfalz J. Bayraktar O. A. Prikryl J. Barkan A. (2009). Site-specific binding of a PPR protein defines and stabilizes 5^′^ and 3^′^ mRNA termini in chloroplasts. *EMBO J.* 28 2042–2052. 10.1038/emboj.2009.121 19424177 PMC2718276

[B44] Pfannschmidt T. Nilsson A. Allen J. F. (1999a). Photosynthetic control of chloroplast gene expression. *Nature* 397 625–628. 10.1038/17624

[B45] Pfannschmidt T. Nilsson A. Tullberg A. Link G. Allen J. F. (1999b). Direct transcriptional control of the chloroplast genes psbA and psaAB adjusts photosynthesis to light energy distribution in plants. *IUBMB Life* 48 271–276. 10690637 10.1080/713803507

[B46] Pfannschmidt T. Schutze K. Brost M. Oelmuller R. (2001). A novel mechanism of nuclear photosynthesis gene regulation by redox signals from the chloroplast during photosystem stoichiometry adjustment. *J. Biol. Chem.* 276 36125–36130. 10.1074/jbc.M105701200 11468291

[B47] Pons T de Jong-Van Berkel Y. (2004). Species-specific variation in the importance of the spectral quality gradient in canopies as a signal for photosynthetic resource partitioning. *Ann. Bot.* 94 725–732. 10.1093/aob/mch197 15374835 PMC4242218

[B48] Porra R. J. Thompson W. A. Kriedemann P. E. (1989). Determination of accurate extinction coefficients and simultaneous-equations for assaying chlorophyll-a and chlorophyll-B extracted with 4 different solvents – verification of the concentration of chlorophyll standards by atomic-absorption spectroscopy. *Biochim. Biophys. Acta* 975 384–394. 10.1016/S0005-2728(89)80347-0

[B49] Prins A. Muchwezi J. Verrier P. Foyer C. (2007). Acclimation of photosynthesis in maize plants to high CO2. *Photosyn. Res.* 91 294–295.

[B50] Prins A. Mukubi J. M. Pellny T. K. Verrier P. J. Beyene G. (2011). Acclimation to high CO2 in maize is related to water status and dependent on leaf rank. *Plant Cell Environ.* 34 314–331. 10.1111/j.1365-3040.2010.02245.x 21054434

[B51] Rintamaki E. Martinsuo P. Pursiheimo S. Aro E. M. (2000). Cooperative regulation of light-harvesting complex II phosphorylation via the plastoquinol and ferredoxin-thioredoxin system in chloroplasts. *Proc. Natl. Acad. Sci. U.S.A.* 97 11644–11649. 10.1073/pnas.180054297 11005828 PMC17254

[B52] Romanowska E. Drozak A. (2006). Comparative analysis of biochemical properties of mesophyll and bundle sheath chloroplasts from various subtypes of C-4 plants grown at moderate irradiance. *Acta Biochim. Pol.* 53 709–719. 17106510

[B53] Romanowska E. Kargul J. Powikrowska M. Finazzi G. Nield J. Drozak A. (2008). Structural organization of photosynthetic apparatus in agranal chloroplasts of Maize. *J. Biol. Chem.* 283 26037–26046. 10.1074/jbc.M803711200 18632664 PMC3258860

[B54] Sambrook J. Fritsch E. F. Maniatis T. (1989). *Molecular Cloning: A Laboratory Manual*. Cold Spring Harbor: Cold Spring Harbor Laboratory Press.

[B55] Smith H. (2000). Phytochromes and light signal perception by plants-an emerging synthesis. *Nature* 407 585–591. 10.1038/35036500 11034200

[B56] Steiner S. Dietzel L. Schröter Y. Fey V. Wagner R. Pfannschmidt T. (2009). The role of phosphorylation in redox regulation of photosynthesis genes psaA and psbA during photosynthetic acclimation of mustard. *Mol. Plant* 2 416–429. 10.1093/mp/ssp007 19825626

[B57] Terashima I. Araya T. Miyazawa S. Sone K. Yano S. (2005). Construction and maintenance of the optimal photosynthetic systems of the leaf, herbaceous plant and tree: an eco-developmental treatise. *Ann. Bot.* 95 507–519. 10.1093/aob/mci049 15598701 PMC4246796

[B58] Tikkanen M. Nurmi M. Kangasjarvi S. Aro E. M. (2008). Core protein phosphorylation facilitates the repair of photodamaged photosystem II at high light. *Biochim. Biophys. Acta* 1777 1432–1437. 10.1016/j.bbabio.2008.08.004 18774768

[B59] Wagner R. Dietzel L. Bräutigam K. Fischer W. Pfannschmidt T. (2008). The long-term response to fluctuating light quality is an important and distinct light acclimation mechanism that supports survival of *Arabidopsis thaliana* under low light conditions. *Planta* 228 573–587. 10.1007/s00425-008-0760-y 18542996

[B60] Walters R. G. (2005). Towards an understanding of photosynthetic acclimation. *J. Exp. Bot.* 56 435–447. 10.1093/jxb/eri060 15642715

[B61] Walters R. G. Rogers J. J. M. Shephard F. Horton P. (1999). Acclimation of *Arabidopsis thaliana* to the light environment: the role of photoreceptors. *Planta* 209 517–527. 10.1007/s004250050756 10550634

[B62] Wittig I. Braun H. P. Schagger H. (2006). Blue native PAGE. *Nat. Protoc.* 1 418–428. 10.1038/nprot.2006.62 17406264

[B63] Zhu X.-G. Long S. P. Ort D. R. (2010). Improving photosynthetic efficiency for greater yield. *Annu. Rev. Plant Biol.* 61 235–261. 10.1146/annurev-arplant-042809-112206 20192734

